# CRISPR-Cas13-Mediated Knockdown of lncRNA-GACAT3 Inhibited Cell Proliferation and Motility, and Induced Apoptosis by Increasing p21, Bax, and E-Cadherin Expression in Bladder Cancer

**DOI:** 10.3389/fmolb.2020.627774

**Published:** 2021-01-18

**Authors:** Zhongfu Zhang, Jieqing Chen, Zhongshuang Zhu, Zhongqing Zhu, Xinhui Liao, Jianting Wu, Jianli Cheng, Xintao Zhang, Hongbing Mei, Guosheng Yang

**Affiliations:** ^1^The Second School of Clinical Medicine, Southern Medical University Affiliated Guangdong Second Provincial General Hospital, Southern Medical University, Guangzhou, China; ^2^Department of Urology, Guangdong Second Provincial General Hospital, Guangzhou, China; ^3^Guangdong Key Laboratory of Systems Biology and Synthetic Biology for Urogenital Tumors, Shenzhen Second People's Hospital, First Affiliated Hospital of Shenzhen University, Shenzhen, China; ^4^Shenzhen Key Laboratory of Genitourinary Tumor, Shenzhen Second People's Hospital, First Affiliated Hospital of Shenzhen University, Shenzhen, China; ^5^Peking University Shenzhen Hospital, Shenzhen, China; ^6^Hong Kong University Shenzhen Hospital, Shenzhen, China; ^7^Shanghai East Hospital, Tongji University School of Medicine, Shanghai, China

**Keywords:** long non-coding RNA, GACAT3, bladder cancer, CRISPR-Cas13, cancer development

## Abstract

The current study is to investigate the expression pattern and biological function of long non-coding RNA Focally gastric cancer-associated transcript3 (GACAT3) in bladder cancer. Real-time quantitative qPCR was used to detect the expression level of GACAT-3 in tumor tissues and paired normal tissues. Human bladder cancer T24 and 5637 cell lines were transiently transfected with specific CRISPR-Cas13 or negative control CRISPR-Cas13. Cell migration, proliferation, and apoptosis were measured by using wound healing assay CCK-8 assay and Caspase-3 ELISA assay, respectively. The expression changes of p21, Bax, and E-cadherin after knockdown of GACAT3 were detected by using Western blot. The results demonstrated that GACAT3 was up-regulated in bladder cancer tissues than that in the paired normal tissues. Inhibition of cell proliferation, increased apoptosis, and decreased motility were observed in T24 and 5637 cell lines transfected by CRISPR-Cas13 targeting GACAT3. Downregulation of GACAT3 increased p21, Bax, and E-cadherin expression and silencing these genes could eliminate the phenotypic changes induced by knockdown of GACAT3. A ceRNA mechanism for GACAT3 was also revealed. By using CRISPR-Cas13 biotechnology, we suggested that GACAT3 may be a novel target for diagnosis and treatment of bladder cancer.

## Introduction

Bladder cancer (BC) is the most common type in malignancy tumors of the urinary system all over the world. The cause of bladder cancer is complex, which include both genetic factors and external environmental factors (Czerniak et al., 2016). Under the action of internal and external factors, the signal networks changed in the bladder epithelial cells lead to the occurrence of bladder cancer. Therefore, the development of bladder cancer is the result of a sophisticated multi-molecule effect (Sathe and Nawroth, 2018). These disrupted molecular networks are the root of the appearance of the malignant phenotypes of bladder cancer. For a long time, we have been trying to find the signal molecules which are located in the center of the signal network and attempt to achieve the goal of interfering with the progression of bladder cancer by acting on these molecules. Although traditional treatment such as surgery, chemotherapy and radiation can treat with bladder cancer to a certain extent, they usually cause severe side effect (Amit and Hochberg, 2010). Thus, it is necessary to develop a new method to deal with bladder cancer specifically.

A growing number body of evidences demonstrated that long non-coding RNAs (lncRNAs) play an important role in the pathological and physiological processes of cells, including the formation of cancers (Huarte, 2015). Compared with other types of RNA such as siRNA, piRNA, or miRNA, the mechanisms of lncRNAs are more sophisticated. Through interaction with RNAs, DNA, or other protein molecules, lncRNAs regulate the expression of many proteins (Jariwala and Sarkar, 2016). LncRNA-DILC inhibits the expression of IL-6 by binding to the IL-6 promoter DNA in hepatocellular carcinoma, thereby inhibiting the growth of cancer stem cells (Wang et al., 2016). In breast cancer, lncRNA-LINP1 was found to serve as an important regulator which enhances DNA-repair activity by interacting with DNA-PKcs and Ku80 proteins (Zhang et al., 2016). Moreover, the dysregulation of hundreds of lncRNAs are closely related to the clinical pathologies of gastric cancer in one previous research (Zhao et al., 2015).

LncRNA-GACAT3 is located on human Chr 2p24.3 and it was reported to be upregulated in some types of cancer tissues, such as gastric cancer (Feng et al., 2018), colorectal cancer (Zhou et al., 2018) and non-small cell lung cancer (Yang et al., 2018). The potential of lncRNA-GACAT3 as an important regulatory point in cancer is gradually developed. Although the role of lncRNA-GACAT3 in regulating cell function has been reported in previous studies, its biological function and molecular mechanism in BC is not clear yet (Shen et al., 2016; Feng et al., 2018; Zhou et al., 2018).

In this study, we found that lncRNA-GACAT3 was upregulated in bladder cancer and can promote malignant phenotypes for the first time. Moreover, we also found that GACAT3 can inhibit cell apoptosis, and promote cell proliferation and migration by downregulating the expression of P21, Bax, and E-cadherin proteins in bladder cancer. These results might provide a novel potential methods for targeted therapy or diagnosis of bladder cancer.

## Methods

### Cell Culture

We purchased the bladder cancer cell lines T24 and 5637 from the Institute of Cell Research, Chinese Academy of Sciences, Shanghai, China. These cells were cultured in DMEM medium which had added 10% fetal bovine serum. We grew the cells in the 37°C atmosphere which contains 5% CO_2_.

### qRT-PCR Assay

The TRIzol reagent (Invitrogen, Carlsbad, CA, USA) was used to extract the total RNAs from the cancer simple tissues and bladder cancer cell lines. The cDNAs all were synthesized from the total extracted RNAs and put into effect with the RevertAid™ First Strand cDNA Synthesis Kit (Fermentas, Hanover, MD, USA). We used the All-in-One™ qPCR Mix (GeneCopoiea Inc, Rockville, MD, USA) to carry out the qRT-PCR assay in our study.

### Western Blot Assay

The antibodies of P21 protein, Bax protein and E-cadherin protein were got from Cell Signaling Technology (Boston, MA, USA). The process of the western blot assay in our study is carried out according to the traditional western blot assay method.

### Cell Counting Kit-8 Assay

We evaluated the level of cell proliferation by using Cell Counting Kit-8 (CCK-8) assay (Beyotime Institute of Biotechnology, shanghai, China). The CCK-8 assay was put into effect according to the instructions of the manufacturer.

### Caspase-3 ELISA Assay

The Caspase-3 ELISA assay kit (R&D, Minneapolis, MN, USA) was used to evaluate the level of cell apoptosis in our study. The operation step of this experiment was performed according to the instructions of manufacturer.

### Wound Healing Assay

The migration level of bladder cancer cells in our study was evaluated by using wound healing assay. We carried out this experiment according to the traditional method.

### Nuclear/Cytoplasmic Fractionation

Nuclear/cytoplasmic fractionation was performed with PARIS Kit (Life Technologies, MA) based on the manufacturer's instructions. After the cell nuclear and cytoplasmic fractionating, the expression level of GACAT3 were determined by RT-qPCR with GAPDH as internal controls, respectively.

### Dual-Luciferase Reporter Assay

The vectors of lncRNA GACAT3 Wild Type or Mutant were constructed and co-transfected with miR-497 mimics or corresponding negative control (miR-NC) into bladder cancer cells. Forty-eight hours after transfection, luciferase activity was measured by using the Dual-Luciferase Reporter Assay System (Promega).

### Statistical Analysis

The different expression level of GACAT3 RNA between bladder cancer tissues and the paired normal tissues were analyzed by using paired samples *t*-test. The GACAT3 RNA expression differences between cancer subgroups were analyzed using independent samples *t*-test. The diversities between different groups in the CCK-8 assay were analyzed by utilizing ANOVA. Cell apoptosis assays and wound healing assays were analyzed using independent samples *t*-test. Pearson's coefficient correlation was used for expression correlation assay. All these statistical analyses were carried out in SPSS (Version 19.0 SPSS Inc.). A *P*-value of < 0.05 was thought to be statistically significant.

## Results

### lncRNA-GACAT3 Was Upregulated in Bladder Cancer Tissues

In our work, a total of 32 sets of urothelial malignant tumor specimens were collected. All these specimens were collected from the patients who diagnosed with bladder cancer and treated with surgical treatment in the Shenzhen second people's Hospital. We conserved the tumor specimens and the normal paired cancer tissues in the liquid nitrogen environment. A written informed consent form is gained from each patient. The study was approved by the first affiliated Hospital of Shenzhen University (Shenzhen, China).

**Figure 1:**
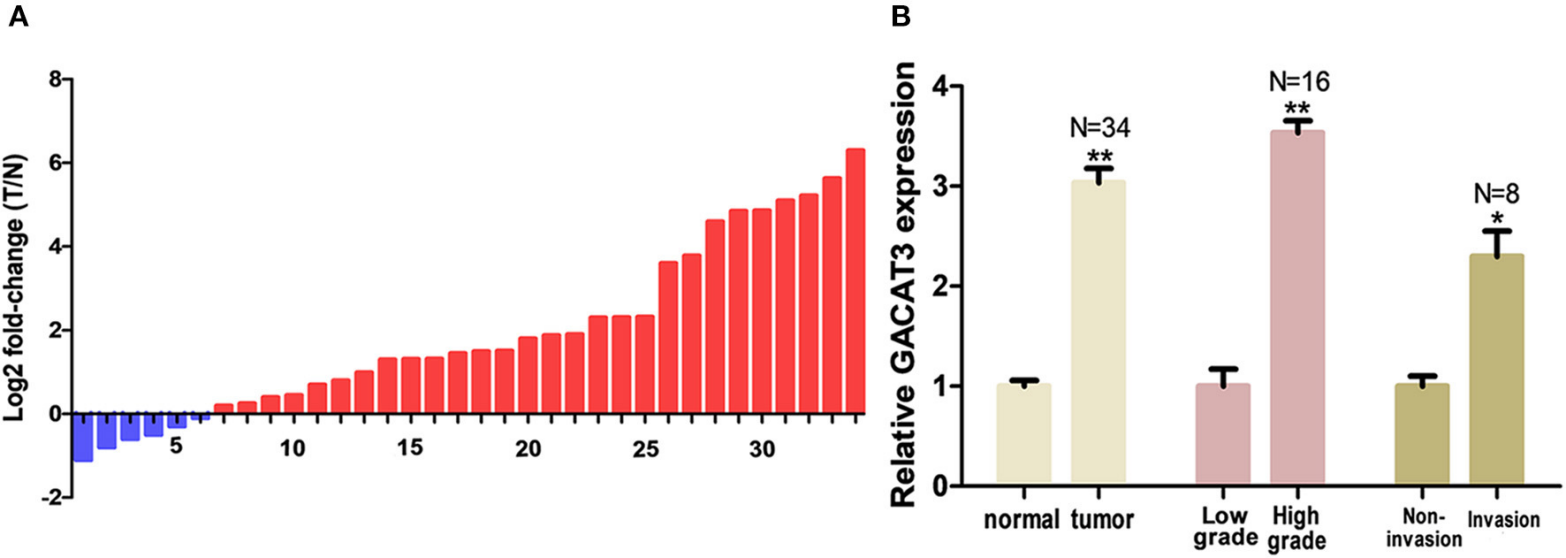
GACAT3 was overexpressed in bladder cancer. Relative GACAT3 expression was detected using Real-Time qPCR. The relative GACAT3 expression value was set as the base value of 1 in each control group. N, number of study population in each group. **(A)** T represents tumor, N represents normal. The heights of the columns in the chart represent the log2-transformed fold changes (tumor/normal) in GACAT3 expression in 36 patients. **(B)** GACAT3 expression levels were higher in tumors than those in normal tissues (**p < 0.01). GACAT3 expression levels were higher in high grade cancers than those in low grade cancers (**p < 0.01). GACAT3 expression levels were higher in invasive cancers than those in non-invasive cancers (*p < 0.05).

The relative expression level of GACAT3 was detected by utilizing the Real-Time qPCR in a total of 32 patients with bladder cancer. The expression fold change of GACAT3 (bladder cancer tissue/matched histologically normal tissue) in each patient is indicated in Figure 1A. The clinical features of this set of patients are displayed in Table 1. GACAT3 was upregulation in bladder cancer compared to the paired normal tissues (Figure 1B). We analyzed expression differences according to grading and staging. The column diagram indicates the relative expression of GACAT3 in each group. GACAT3 expression levels were higher in high grade tumors than in low grade tumors (Figure 1B). GACAT3 expression levels were higher in invasive tumors than in non-invasive tumors (Figure 1B).

**Table 1:** Clinical features of patients with bladder cancer.

**No**.	**Sex**	**Age**	**Grade**	**Stage**	**Surgery**	**No**.	**Sex**	**Age**	**Grade**	**Stage**	**Surgery**
1	M	57	Low	T2N0M0	Partial	18	M	61	Low	T1N0M0	Partial
2	M	64	Low	T1N0M0	Partial	19	M	64	High	T2N0M0	Radical
3	M	51	Low	T2N0M0	Partial	20	M	61	Low	T1N0M0	Partial
4	M	64	High	T3N0M0	Radical	21	M	54	High	T2N0M0	Radical
5	M	65	Low	T2N0M0	Partial	22	M	58	High	T2N0M0	Radical
6	M	58	Low	T1N0M0	Partial	23	M	50	High	T3N0M0	Radical
7	M	53	High	T2N0M0	Radical	24	M	42	High	T2N0M0	Radical
8	M	61	Low	T2N0M0	Partial	25	M	58	High	T2N0M0	Radical
9	M	41	Low	T1N0M0	Partial	26	M	57	High	T3N0M0	Radical
10	M	54	High	T2N0M0	Radical	27	F	58	High	T3N0M0	Radical
11	M	51	High	T2N0M0	Radical	28	F	56	High	T3N0M0	Partial
12	M	55	Low	T2N0M0	Partial	29	F	51	Low	T1N0M0	Partial
13	M	55	Low	T2N0M0	Partial	30	F	52	Low	T1N0M0	Partial
14	M	73	High	T2N0M0	Radical	31	F	51	High	T3N0M0	Partial
15	M	55	Low	T1N0M0	Partial	32	F	52	High	T3N0M0	Radical
16	M	50	Low	T1N0M0	Partial	33	F	43	High	T3N0M0	Radical
17	M	57	High	T2N0M0	Radical	34	F	69	High	T3N0M0	Radical

*No., patient number; M, male; F, female; Age, years old; Grade, the 2004 WHO classification; Stage, AJCC TNM classification; Radical, radical cystectomy; Partial, partial cystectomy*.

### Specific CRISPR-Cas13 Down-Regulated the Expression of lncRNA-GACAT3

Bladder cancer cell lines T24 and 5637 were grown and transfected with CRISPR-Cas13 or negative control CRISPR-Cas13. Forty-eight hours after transfection, the GACAT3 RNA expression levels were analyzed by Real-Time qPCR. The inhibitory rate (GACAT3 CRISPR-Cas13/negative control CRISPR-Cas13) was 80.23 ± 2.19% in T24 cells and 79.31 ± 4.81% in 5637 cells, respectively. Data are shown as mean ± SD. Each experiment in both cell lines was performed in triplicate for three independent times.

### Knockdown of lncRNA-GACAT3 Inhibited Cell Proliferation

**Figure 2:**
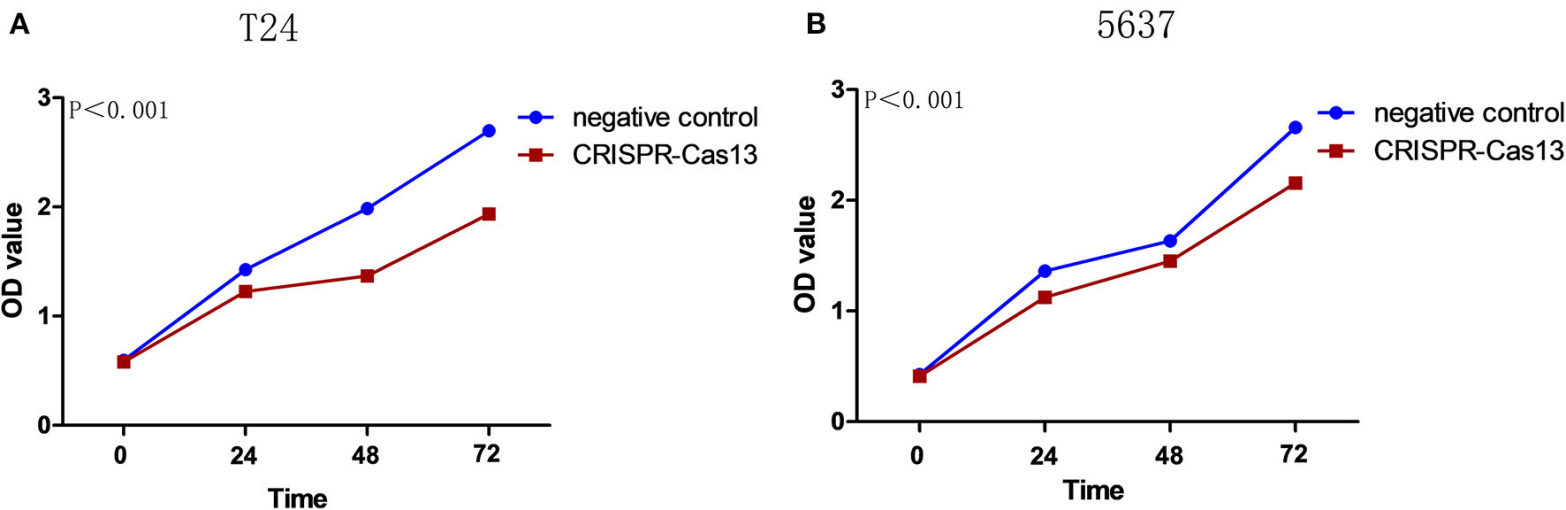
Knockdown of GACAT3 inhibited cell proliferation. Cell proliferation was measured by CCK-8 assay. After transfection of CACAT3 CRISPR-Cas13 or negative control CRISPR-Cas13, OD values were measured and converted to cell numbers. ANOVA was used for the comparison of curves of cell proliferation. **(A)** Inhibition of cell proliferation was observed in bladder cancer 5637 cells (**p < 0.01). **(B)** Inhibition of cell proliferation was observed in bladder cancer T24 cells (**p < 0.01). Data are shown as mean ± SD. Each experiment in both cell lines was performed in double for three independent times.

Bladder cancer T24 and 5637 cells were transfected with GACAT3 related CRISPR-Cas13 or negative control CRISPR-Cas13 and the cell proliferation rates of bladder cancer cells were determined by CCK-8 assay. Cell proliferation was inhibited in both T24 cells (Figure 2A) and 5637 cells after knockdown of GACAT3 (Figure 2B).

### Knockdown of lncRNA-GACAT3 Induced Apoptosis and Inhibited Cell Migration

**Figure 3:**
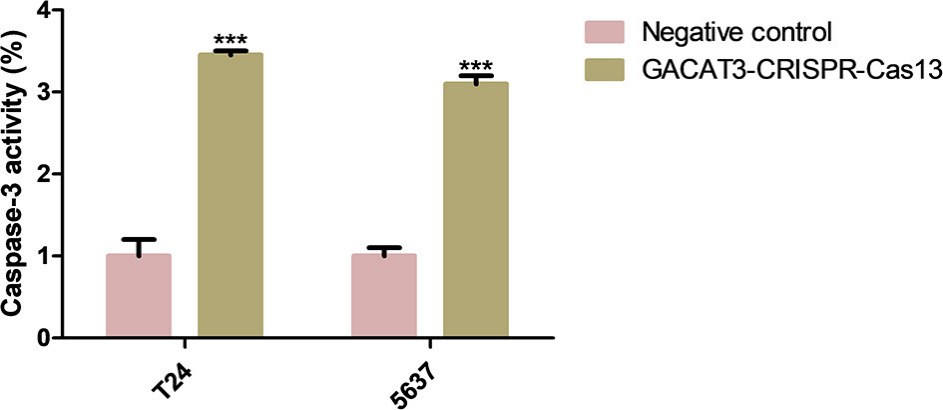
Knockdown of GACAT3 induced apoptosis. Forty-eight hours after transfection of GACAT3 CRISPR-Cas13 or negative control CRISPR-Cas13, the cell apoptosis changes were determined by ELISA. Cell apoptosis induction was observed in GACAT3 CRISPR-Cas13-transfected bladder cancer 5637 (***p < 0.01) and T24 (***p < 0.01) cells using ELISA. Data are shown as mean ± SD. Each experiment in both cell lines was performed in triplicate for three independent times.

**Figure 4:**
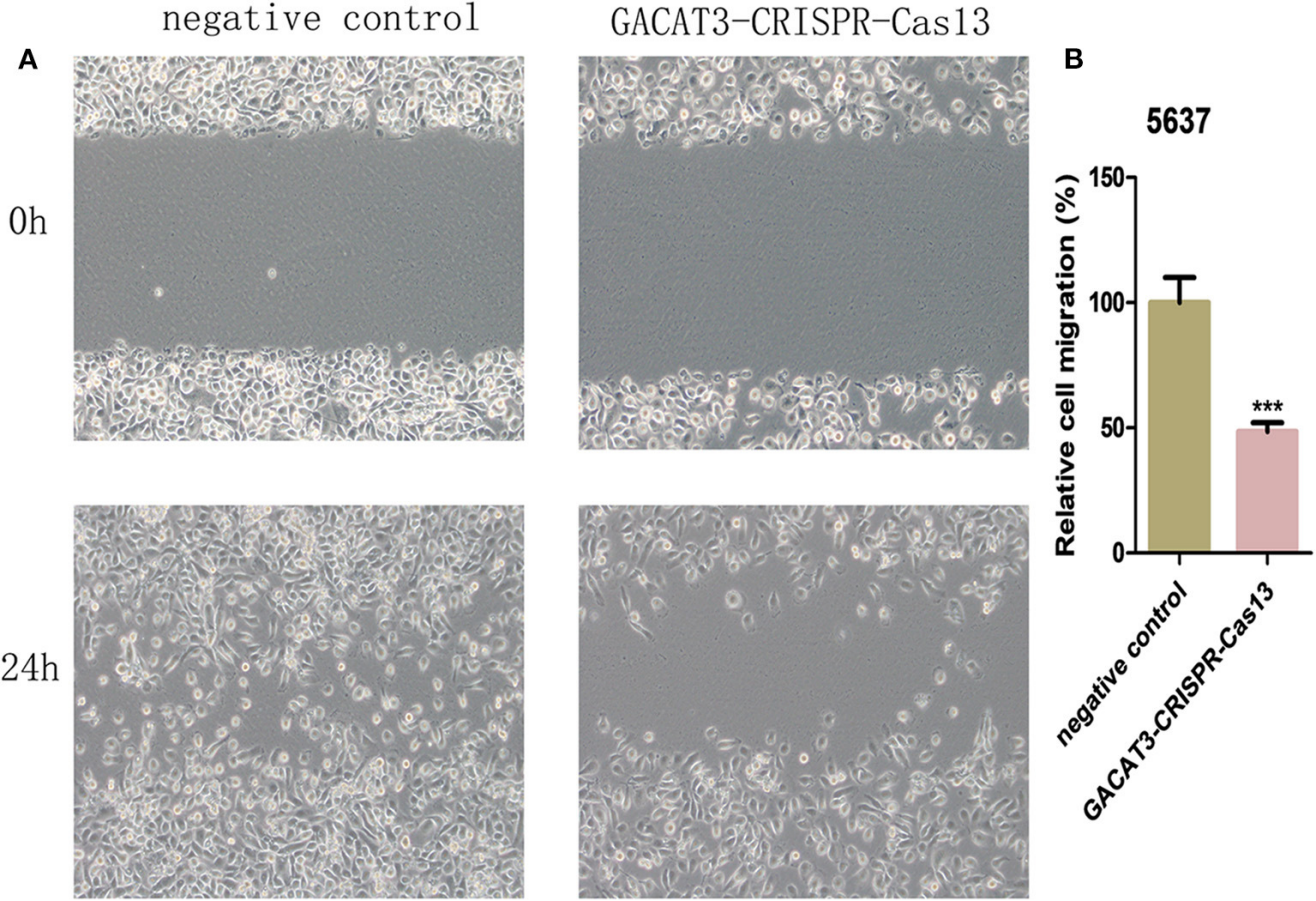
Knockdown of GACAT3 decreased cell motility. After transfection of GACAT3 CRISPR-Cas13 or negative control CRISPR-Cas13, wound healing assay was used to detect cell motility changes in bladder cancer cells. **(A)** Representative images of wound healing assay in 5637 and T24 cells. **(B)** Decreased cell motility was observed in 5637 and T24 cells. Data are indicated as mean ± SD. Each experiment in both cell lines was performed in triplicate for three independent times.

Bladder cancer T24 and 5637 cell lines were transfected with GACAT3 related CRISPR-Cas13 or negative control CRISPR-Cas13. Forty-eight hours after transfection, the cell apoptosis and migration of bladder cancer T24 and 5637 cells were determined using ELISA (Figure 3) and wound healing analysis (Figures 4A,B). Induced cell apoptosis and suppressed cell migration were observed in both bladder cancer cell lines after knockdown of GACAT3.

### Knockdown of GACAT3 Increased p21, Bax, and E-Cadherin Protein Expression

**Figure 5:**
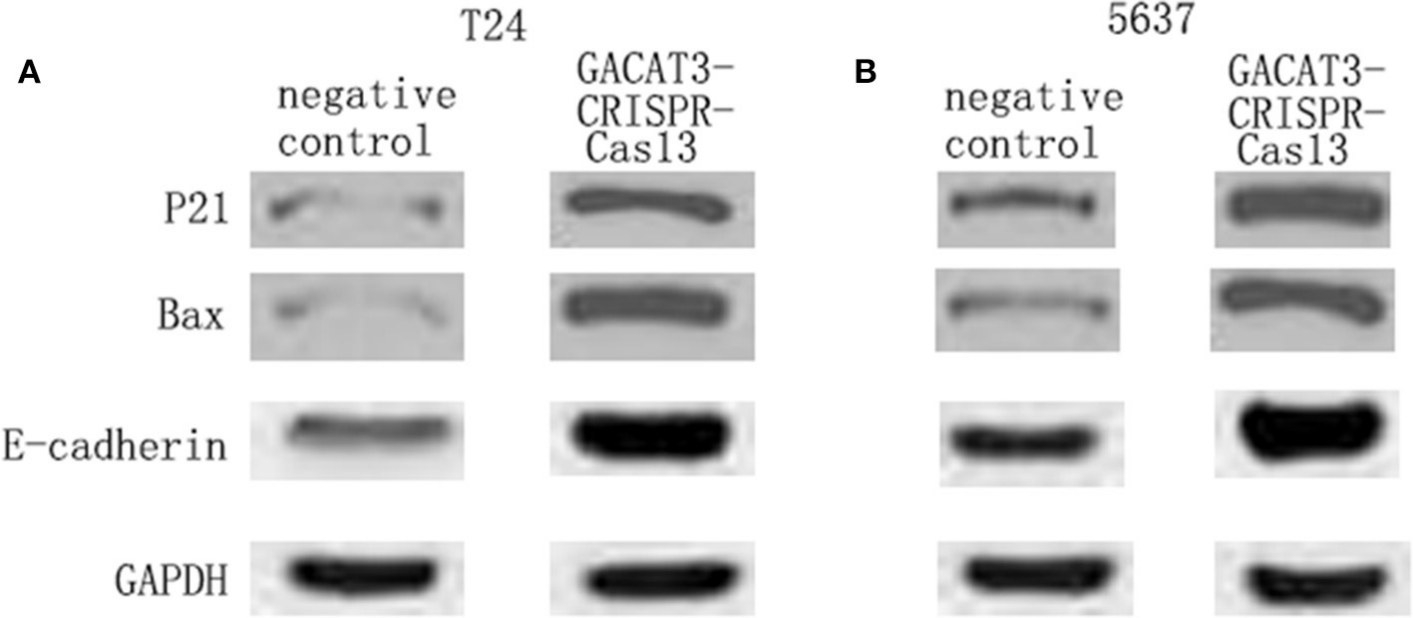
Knockdown of GACAT3 increased p21, Bax, and E-cadherin protein expression. After transfection of GACAT3 CRISPR-Cas13 or negative control CRISPR-Cas13, western blot assay was used to detect expression changes of p21, Bax, and E-cadherin in bladder cancer cells. **(A)** Representative images of western blot assay in 5637 cells. **(B)** Representative images of western blot assay in T24 cells.

**Figure 6:**
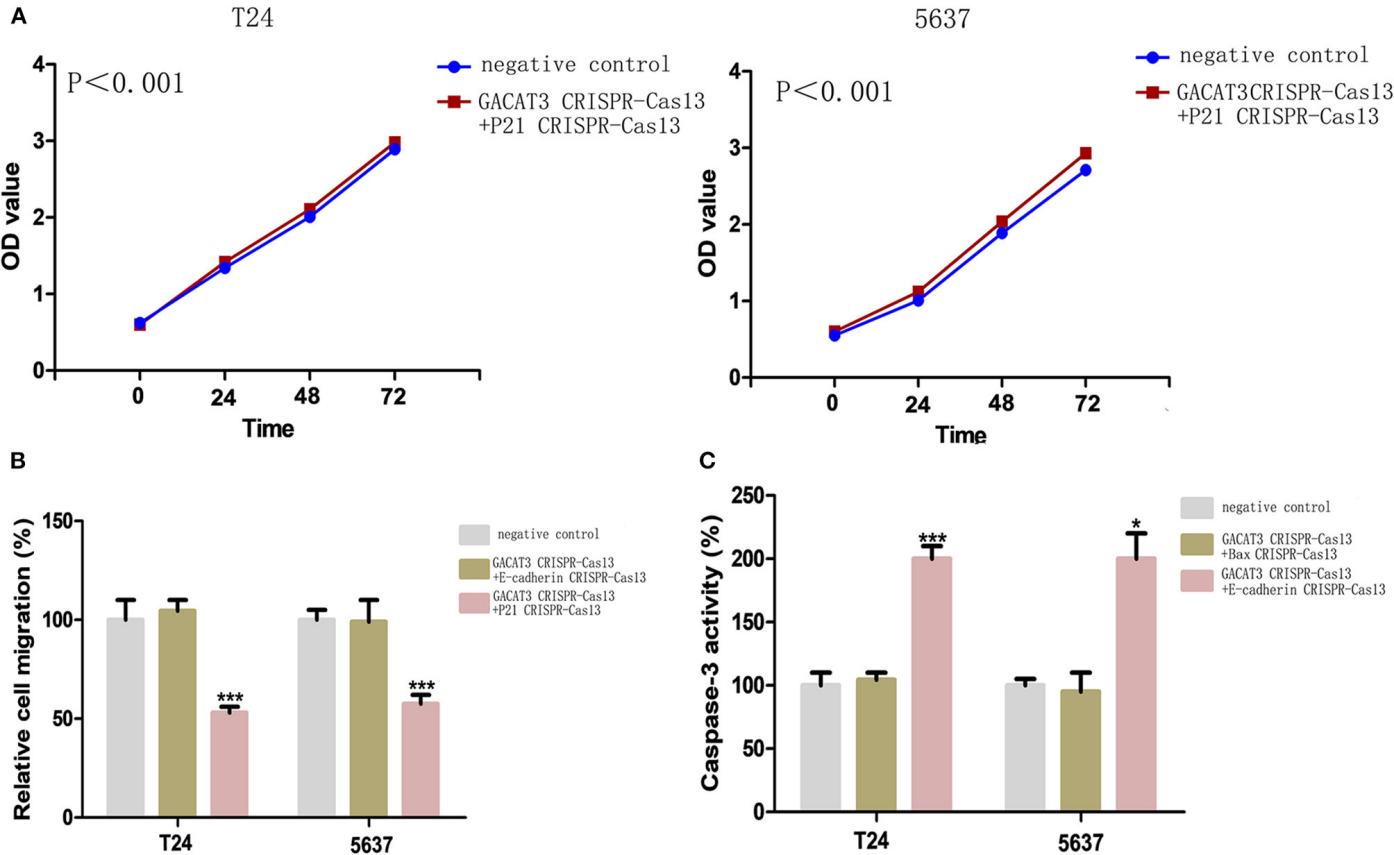
Knockdown of GACAT3 induced cellular phenotypic changes *via* p21, Bax, and E-cadherin regulation. After transfection of CRISPR-Cas13, CCK-8 assay, wound healing assay, and ELISA assay were used to detect cell proliferation, migration and apoptosis, respectively. **(A)** p21 CRISPR-Cas13 eliminated the proliferation inhibition effects induced by GACAT3 CRISPR-Cas13 in T24 and 5637 (*p* < 0.05). **(B)** E-cadherin CRISPR-Cas13 eliminated the migration inhibition effects induced by GACAT3 CRISPR-Cas13 in T24 and 5637, whereas p21 CRISPR-Cas13 has no such function (***p < 0.001). **(C)** Bax CRISPR-Cas13 eliminated the apoptosis-promoting effects induced by GACAT3 CRISPR-Cas13 in T24 and 5637, whereas E-cadherin CRISPR-Cas13 has no such function (*p < 0.05).

To investigate the potential bio-markers that induce the above phenotypic changes after knockdown of GACAT3, we used western blot assay to determine the protein levels of p21, Bax, and E-cadherin that are well-known for bladder cancer development. GACAT3 CRISPR-Cas13 significantly up-regulated the expression of p21, Bax, and E-cadherin at protein levels in T24 (Figure 5A) and 5637 cells (Figure 5B). To further verify the functional roles of these markers in GACAT3 knockdown experiments, we also performed double knockdown using two CRISPR-Cas13. p21 CRISPR-Cas13 eliminated the proliferation inhibition effects induced by GACT3 CRISPR-Cas13 in T24 and 5637 (Figure 6A). E-cadherin CRISPR-Cas13 eliminated the migration inhibition effects induced by GACAT3 CRISPR-Cas13 in T24 and 5637 (Figure 6B). Bax CRISPR-Cas13 eliminated the apoptosis-promoting effects induced by GACAT3 CRISPR-Cas13 in T24 and 5637 (Figure 6C). p21 CRISPR-Cas13 did not reverse the migration inhibition effects (Figure 6B) and apoptosis-promoting effects (Figure 6C) induced by GACAT3 CRISPR-Cas13 in these cells, possibly because p21 could not induce cell death or inhibit cell migration on its own.

### lncRNA GACAT3 Was Predominantly Distributed in Cytoplasm and Acted as a Sponge for miR-497

**Figure 7:**
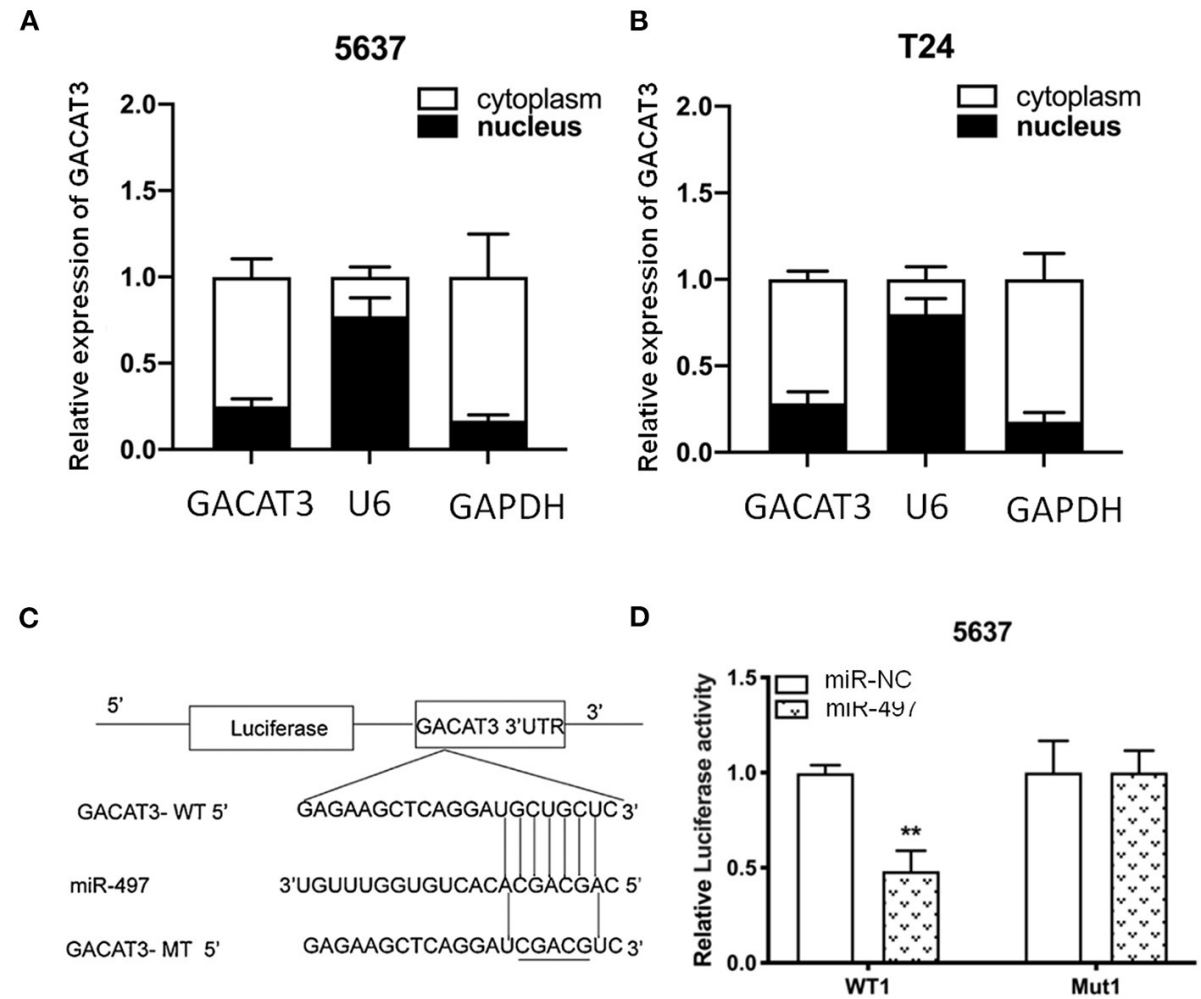
lncRNA GACAT3 directly interacted with miRNA-497. **(A)** Nuclear/cytoplasmic fractionation assay revealed that lncRNA GACAT3 primarily distributed in cytoplasm of Bca 5637 cells. **(B)** Nuclear/cytoplasmic fractionation assay revealed that lncRNA GACAT3 primarily distributed in cytoplasm of Bca T24 cells. **(C)** Bioinformatics analysis predicted the probable sequences of lncRNA GACAT3 that are complementary to the seed sequence of miR-497. **(D)** Dual-luciferase assay showed that co-transfection of lncRNA GACAT3 and miR-497 significantly inhibited luciferase activity (**p < 0.01).

To further explore the mechanism how lncRNA GACAT3 modulates tumorigenesis, we used nuclear/cytoplasmic fractionation assay. The experimental results showed that lncRNA GACAT3 was primarily distributed in cytoplasm of Bca cells (Figure 7A). miRDB database (http://mirdb.org/miRDB/) predicted that miR-497 has a sequence complementary to that of GACAT3 (Figure 7B). The potential sequence of lncRNA GACAT3 which was predicted complementary to the seed sequence of miR-497 were mutated and used to construct dual-luciferase reporter vector. Dual-luciferase reporter assay showed that co-transfection of lncRNA GACAT3-WT and miR-515-5p significantly inhibited luciferase activity than that of control group (Figures 7C,D). It demonstrated the ceRNA mechanism for GACAT3.

## Discussion

Recent research indicated that human cancers are caused by dysregulation of a large number of lncRNAs. It has been believed that lncRNAs have great clinical potential as a group of cancer biomarkers and therapeutic targets (Sahu et al., 2015; Sun and Kraus, 2015). For example, lncRNAs MALAT1 (Tian and Xu, 2015), SUMO1P3 (Zhan et al., 2016), and CCAT2 (Li et al., 2016) have been reported to promote cell proliferation and suppress cell apoptosis in bladder cancer. As a recently discovered lncRNA, several previous works were conducted to characterize the oncogenic properties of GACAT3. It may represent a therapeutic target in cancers and increases the transcription of genes including p21 *via* stabilization of BMI1 (Hu et al., 2014; Jeong et al., 2016). It has been reported that p21 is a gene that involved in several processes, such as cell proliferation and apoptosis. Knockdown of GACAT3 stimulated cell-cycle arrest and senescence. GACAT3 plays an important role in cell-cycle progression and was associated with aggressive tumor behavior. These data demonstrated that GACAT3 may be a cancer driver during tumor development.

Since GACAT3 displayed striking oncogenic activity in previous studies (Feng et al., 2018), it is intriguing to explore its biological function in bladder cancer. With this aim, in this study, we found that GACAT3 was overexpressed in bladder cancer compared to matched normal tissue. High level expression of GACAT3 was associated with high grade and stage bladder cancer. The differential expression patterns of GACAT3 between bladder cancer and control and the association of GACAT3 with clinicopathological features suggest that long non-coding RNA GACAT3 emerges as a novel player in the development and progression of the bladder cancer.

To understand the possible impacts of GACAT3 on bladder cancer, we determined the changes in cell proliferation, apoptosis and motility induced by knockdown of GACAT3 in bladder cancer by using CRISPR-Cas13 biotechnology, which has a better targeting performance than that of siRNA/shRNA. Inhibition of cell proliferation, increased apoptosis, and decreased motility were observed in GACAT3 CRISPR-Cas13-transfected bladder cancer T24 and 5637 cell lines.

To investigate the potential pathways that induce the above phenotypes, we also performed western blot assay and found that knockdown of GACAT3 increased p21, Bax, and E-cadherin protein expression. This result was consistent with the previous studies which indicated that p21 is one GACAT3 downstream target. In other studies on bladder cancer, inactivation of p21 was also shown to promote tumorigenesis and cell proliferation (Tang et al., 2015). Bax, an important homolog of Bcl-2, is a promoter of cell apoptosis and serves as an independent parameter to envisage clinical outcome for patients with bladder cancer (Golestani Eimani et al., 2014; Liu et al., 2016). E-cadherin is a cell-cell junction protein that is frequently absent during the migration of bladder cancer cells (Liu et al., 2014; Zhao et al., 2014). Using double knockdown experiments, we further showed that the expression changes of these bio-markers can at least partly explain the phenotypic changes after knockdown of GACAT3. GACAT3 may associate with the epigenetic repressors, thus regulating the transcription of Bax or E-cadherin. Further studies demonstrated that miR-497 is a direct target of GACAT3, which indicated a ceRNA mechanism.

These findings suggest that GACAT3 functions as an oncogene in carcinogenesis of bladder cancer. Targeting GACAT3 may be a promising approach to the treatment of bladder cancer. More works will be needed to determine the potential molecular mechanism of GACAT3 in the regulation of Bax and E-cadherin in bladder cancer. CRISPR-Cas13-based therapies (Gao et al., 2020; Li et al., 2020) that target GACAT3 are also should under intensive investigation.

## Data Availability Statement

The original contributions presented in the study are included in the article/Supplementary Materials, further inquiries can be directed to the corresponding author.

## Author Contributions

ZhongfZ, JC, ZhongsZ, ZhongqZ, XL, JW, JC, XZ, and HM performed experiments and conducted data analysis. GY supervised the project. All authors contributed to the article and approved the submitted version.

## Conflict of Interest

The authors declare that the research was conducted in the absence of any commercial or financial relationships that could be construed as a potential conflict of interest.
